# Reduced interference in working memory following mindfulness training is associated with increases in hippocampal volume

**DOI:** 10.1007/s11682-018-9858-4

**Published:** 2018-03-17

**Authors:** Jonathan Greenberg, Victoria L. Romero, Seth Elkin-Frankston, Matthew A. Bezdek, Eric H. Schumacher, Sara W. Lazar

**Affiliations:** 1 Department of Psychiatry, Massachusetts General Hospital, Boston, MA USA; 2 Harvard Medical School, Boston, MA USA; 3 Charles River Analytics, Cambridge, MA USA; 4 School of Psychology, Georgia Institute of Technology, Atlanta, GA USA

**Keywords:** Mindfulness, Proactive interference, Working memory, Hippocampus, MRI

## Abstract

Proactive interference occurs when previously relevant information interferes with retaining newer material. Overcoming proactive interference has been linked to the hippocampus and deemed critical for cognitive functioning. However, little is known about whether and how this ability can be improved or about the neural correlates of such improvement. Mindfulness training emphasizes focusing on the present moment and minimizing distraction from competing thoughts and memories. It improves working memory and increases hippocampal density. The current study examined whether mindfulness training reduces proactive interference in working memory and whether such improvements are associated with changes in hippocampal volume. 79 participants were randomized to a 4-week web-based mindfulness training program or a similarly structured creative writing active control program. The mindfulness group exhibited lower proactive interference error rates compared to the active control group following training. No group differences were found in hippocampal volume, yet proactive interference improvements following mindfulness training were significantly associated with volume increases in the left hippocampus. These results provide the first evidence to suggest that (1) mindfulness training can protect against proactive interference, and (2) that these benefits are related to hippocampal volumetric increases. Clinical implications regarding the application of mindfulness training in conditions characterized by impairments to working memory and reduced hippocampal volume such as aging, depression, PTSD, and childhood adversity are discussed.

## Introduction

Proactive interference occurs when previously learned material impairs detainment of newer material (Abel and Bäuml 2014; Keppel and Underwood 1962; Loosli et al. 2016). It has been suggested that the loss of information from working memory would be minimal or nonexistent without such interference (Jonides and Nee 2006; Keppel and Underwood 1962). Overcoming proactive interference is critical for high-level cognitive processing such as reasoning and problem solving (Jonides and Nee 2006). However, despite the central role of overcoming proactive interference in cognitive performance, little is known about whether and how this ability can be improved.

Overcoming proactive interference involves successfully distinguishing older from newer material in memory. The hippocampus plays a key role in making such distinction, both in long term (Barredo et al. 2015; Caplan et al. 2007; Douchamps et al. 2013; Kirwan et al. 2007; Schweinsburg et al. 2010; Yassa and Stark 2011), and working memory (Griffin 2015; Leszczynski 2011; Nauer et al. 2015; Öztekin et al. 2010; Ranganath and D’Esposito 2001; Stern et al. 2001). Previous research demonstrated that proactive interference in working memory involves functional activation in both the hippocampus (Öztekin et al. 2008) and the ventrolateral prefrontal cortex (VLPFC), primarily in the left hemisphere (Jonides and Nee 2006; Loosli et al. 2016; Nee et al. 2007), though each region plays a different role in the resolution of proactive interference. Specifically, the left VLPFC reflects engagement of controlled retrieval operations (Badre and Wagner 2007), regardless of retrieval success, whereas the hippocampus was shown to be more directly involved in successful proactive interference resolution (Öztekin et al. 2008).

Given that proactive interference stems from past and currently-irrelevant material and is associated with the hippocampus, we posit that mindfulness training, which emphasizes present moment awareness, may be an effective way to reduce proactive interference, in part through its impact on hippocampal structure. Mindfulness involves regulation of attention to the experience of the present moment in a non-judgmental way (Bishop et al. 2004; Kabat-Zinn 1990). Mindfulness training has been shown to improve working memory (Jha et al. 2010; Mrazek et al. 2013; Quach et al. 2015; van Vugt and Jha 2011), improve encoding of novel material (Bonamo et al. 2015), and increase autobiographical memory specificity (Heeren et al. 2009; Williams et al. 2000).

In addition to its impact on memory, mindfulness training has been documented to impact hippocampal structure. Magnetic resonance imaging (MRI) studies of 8-week mindfulness training programs have revealed increases in hippocampal grey matter density among healthy individuals (Holzel et al. 2011) and adults with Parkinson’s disease, as well as reduced hippocampal atrophy among adults with mild cognitive impairment (Wells et al. 2013) following training. Our group has found similar increases in hippocampal volume following both 8-week meditation and yoga programs compared to a control group of psychoeducation. These results following mindfulness training are supported by cross sectional studies indicating greater hippocampal gray matter among long-term meditators compared to non-meditators (Hölzel et al. 2008; Luders et al. 2013a, b). However, despite the documented impact of mindfulness training on hippocampal structure, the relation between such changes in hippocampal structure following training and improvement in cognitive performance has yet to be examined.

The hippocampus is our current region of interest, due to the its demonstrated malleability following mindfulness programs and other behavioral training programs lasting several weeks (Thomas et al. 2016) or even mere hours (Sagi et al. 2012). Other regions associated with proactive interference such as the VLPFC have not shown such structural malleability following similar training (see Fox et al. 2014; Gotink et al. 2016 for reviews) and are not the focus of the current work.

In this study, we contrasted the effects of a 4-week web-based mindfulness program with a similarly structured 4-week web-based creative writing active control program on proactive interference in working memory. In addition to completing a task that measures proactive interference, a subset of participants underwent MRI scanning before and after the programs. We hypothesized that (a) individuals undergoing mindfulness training will exhibit greater improvements in proactive interference compared to individuals in an active control group; (b) individuals undergoing mindfulness training will exhibit greater increases in hippocampal volume compared to individuals in an active control group; and (c) behavioral improvements in proactive interference following mindfulness training will be associated with increases in hippocampal volume.

## Methods

### Participants

**Fig. 1:**
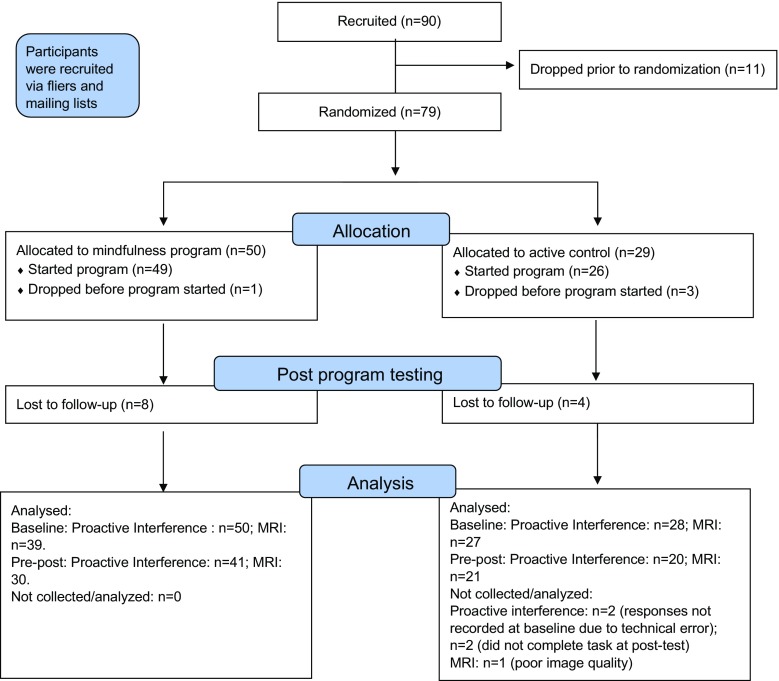
Participant flow

Participant flow is depicted in Fig. 1. Ninety Participants aged 18–50 were recruited via fliers and various mailing lists. Seventy-nine were randomized and 75 started their assigned program (enrolled). Since this study was a pilot for a larger multisite trial that used moderately high-functioning participants, inclusion criteria were: scoring within top 25th percentile on the SAT (minimal score of 580 verbal, 610 math, 570 writing), OR if SAT scores were not available: already completed at least 2 years at a 4-year college and currently enrolled, OR completed a degree at a 4-year college. Exclusion criteria included any neurologic or psychiatric diseases within the past year as self-reported during the phone screen, taking any other psychiatric medication other than a single antidepressant, PTSD symptomology as assessed by the PTSD checklist-civilian (PCL-C; Ruggiero et al. 2003), presence of any MRI contra-indicators (e.g. metallic implants, claustrophobia), left-handedness and having more than 3 previous meditation classes or more than 20 mind–body classes such as yoga or Tai-Chi. To minimize prior familiarity with the test or the expected effects of the interventions, students enrolled in a psychology academic program were excluded. Since the training programs and materials were administered via the web, all participants were additionally required to have reliable internet access with a video camera. Due the rapidly growing clinical interest in the effects of mindfulness training (Chiesa et al. 2011; Chiesa and Serretti 2011; Keng et al. 2011), participants were randomized to the mindfulness or creative writing programs on a 2:1 ratio respectively in order to maximize statistical power to determine the relationships between our measures of interest specifically among the mindfulness group. A total of nine four-week programs (6 mindfulness programs, 3 creative writing programs) were delivered in random order. Participants were assigned to the next available program after completing baseline testing. Assignment to a given program continued until either group size reached a maximum of 15 participants or the first class of that program commenced. Participants were only informed which program they were receiving at the start of their first class. In total, 50 participants were assigned to the mindfulness program, and 29 to the creative writing program. Groups did not differ in age (*t*(77) = 0.55, *p* = .96), gender (*p* = .56, Fisher’s exact test), race (*p* = .19, Fisher’s exact test when examining White and Asian participants, since other races had too few cases to reliably include in the analysis), or education level (*p* = .27, Fisher’s exact test when examining participants who completed 2 or 4 year college, or graduate school, since participants with lower education levels had too few cases to reliably include in the analysis; see Table 1).

**Table 1:** Demographic characteristics of randomized participants

	Mindfulness	Control	Group difference *p* value
	n = 50	n = 29	
Gender (Female %)	70%	69%	*p* = .*56*
Age (Mean, SD)	27.32 (5.59)	27.24 (6.92)	*p* = .96
Race			*p* = .19
White	66%	62%	
Asian	20%	34%	
Hispanic	8%	0%	
Black	6%	4%	
Education			*p* = .27
Some college or less	8%	7%	
2 or 4 year college	52%	62%	
Graduate school	40%	31%	

Due to budget constraints, only the first 67 recruited participants underwent MRI scanning (39 mindfulness group, 28 active control group). The only factor determining receiving MRI was the time of recruitment. Importantly, these last participants who did not receive MRI did not differ from the rest of the sample in any of the examined variables, including age (*t*(76) = 0.11, *p* = .91), gender (*p* = .55, Fisher’s exact test) or baseline proactive interference rates (maximal *t* = 0.76, *p* = .45).

Figure 1 summarizes the collected and available data. Participants who completed post-testing did not differ from those who dropped out in their assigned group (*p* = .15, Fisher’s exact test), baseline proactive interference (maximal *t* = 0.66, *p* = .51), baseline hippocampal volume (maximal *t* = 1.09, *p* = .28), age (*t* = 0.52, *p* = .60) or gender (*p* = .52, Fisher’s exact test), suggesting that missing data following attrition can be considered missing at random. Participants who practiced less than once a week on average were deemed non-adhering (6 participants from the control group, 1 from the mindfulness group). The main results are reported both including and excluding these participants. Participants were remunerated up to $155 for completing the study.

### Training programs

Both programs consisted of four weekly hour-long classes delivered via Zoom.us web-based software, which enables participants to see and communicate with the teacher and other group members via webcam. Classes were recorded so that individuals who missed a class could view it later. Participants in both programs were instructed to practice on their own for 30 min five times a week via a secure online web-portal which provided either guided meditation recordings or writing prompts depending on group assignment. The portal recorded the amount of time students spent practicing. The practice frequency and duration were designed to meet requirements for a future workplace implementation of the program.

#### Mindfulness program

The program was led by a trained mindfulness teacher with over 15 years of experience teaching mindfulness meditation. Thirty minutes of each class were devoted to mindfulness practice, while the remainder consisted of instruction on how to practice, and question-and-answer sessions to support a more in-depth understanding of the concept of mindfulness. The first 2 weeks were devoted to focused-attention meditation in which attention was focused solely on the breath or body sensations (body scan); the second 2 weeks were devoted to “open monitoring”, in which attention was centered on the present experience without predetermining a specific object of focus. Throughout the program an emphasis was placed on identifying when the mind wandered, accepting distractors as transient phenomena to be noticed and observed rather than acted or elaborated upon, and continuously re-establishing awareness of the sensations, thoughts, and feelings as they unfold in the present moment.

#### Creative writing program

Creative writing was chosen as a credible control program which could be provided via a similar format and with the same teacher-support, group setting, active engagement, demand characteristics, and home practice as the mindfulness program. Moreover, creative writing has similarly been found to reduce stress and improve mental (King et al. 2013; Penman et al. 1999), and physical (Lowe 2006) health. However, to our knowledge there is no evidence indicating that it leads to alterations in brain structure or working memory. The creative writing program was led by a professional writing tutor. Writing exercises consisted of writing short essays in response to a photo or short text extracted from Wikipedia.org. Participants were instructed to write material in the format of a daily newspaper article for the first 2 weeks then in an academic scholar format for the final 2 weeks. Didactic instruction included effective writing techniques, concise written communication, and paragraph structure, as well as question-and-answer periods.

### Behavioral proactive interference task

**Fig. 2:**
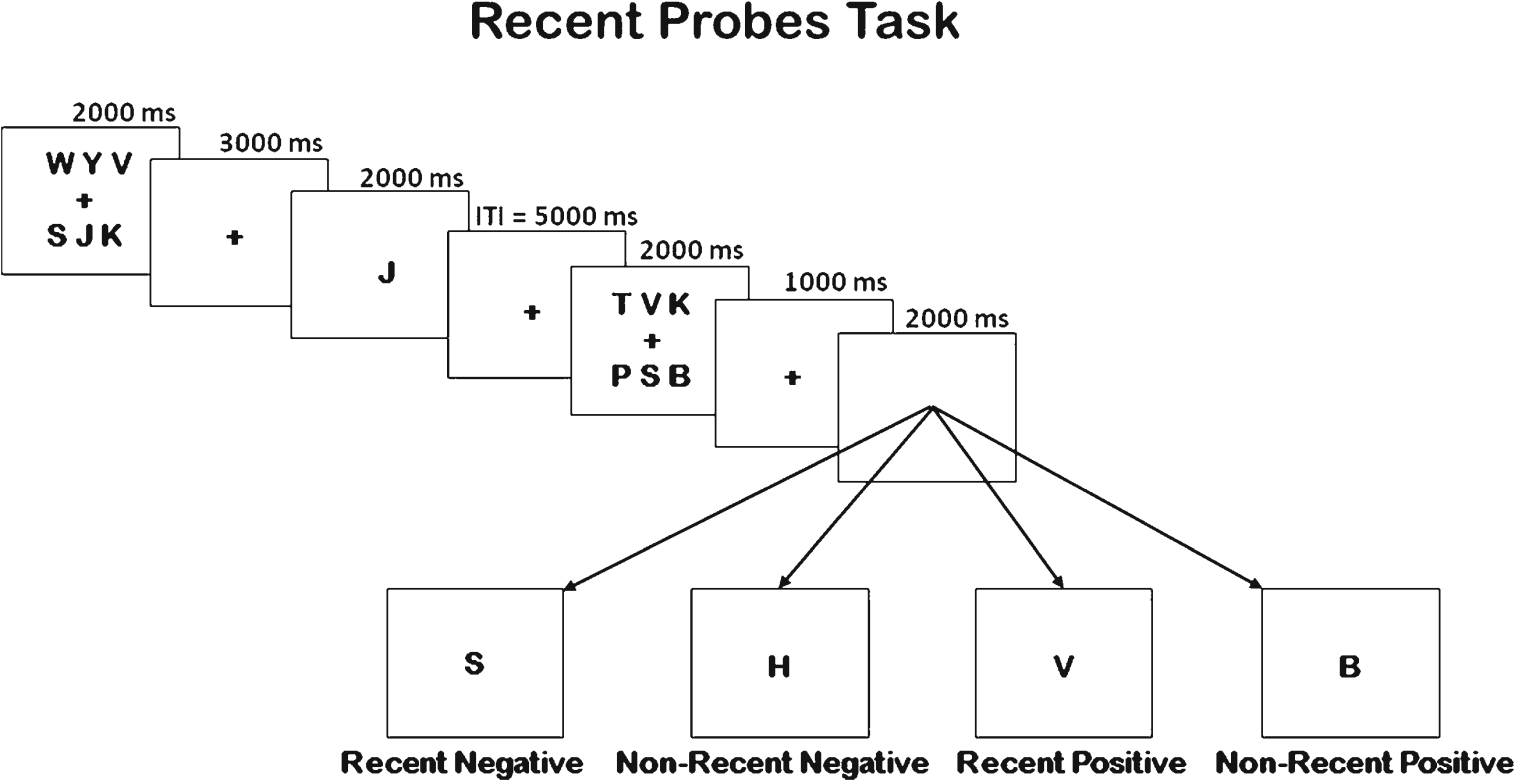
Illustration of the recent probes task

Proactive interference was measured by the Recent Probes task, one of the most widely used measures of proactive interference in working memory (Atkins et al. 2011; Nee et al. 2007). The task was similar to that used and described by Nee et al. (2007). It was administered via E-Prime 2 (Psychological Software Tools, Inc. 2005) and run on Lenovo laptop computers with Intel Core i-5 processors and 19.1″ monitors. The task consisted of a total 144 of trials, which took approximately 20 min to administer. In each trial a red fixation cross appeared for 1 s, followed by a target set of 6 letters presented for 2 s (Fig. 2) which participants were asked to commit to memory. The letters were then replaced by a fixation cross for a delay period of 3 s. Next, a single letter probe appeared for 2 s, and participants were asked to press the “up” arrow if the probe matched the previous target set or the “down” arrow if it did not. Following an inter-trial-interval (ITI) of 1 s (2 s including the following trial’s fixation cross), the next trial began. Each target set was composed of three letters that were present in the previous target set and three novel letters so that half the probes matched the previous target set and half the probes did not. There were four kinds of probes, each occurring in 25% of trials: “recent negative” probes appeared in the previous but not the current target set, “non-recent negative” did not appear in the previous or current target sets, “recent positive” probes appeared in the previous and current target sets, and “non-recent positive” probes appeared in the current but not previous target sets (Fig. 2).

Proactive interference was calculated by subtracting error rates and reaction times (RT) on “non-recent negative” trials from those on “recent-negative trials”. The degree to which old information can facilitate learning newer material is an additional measure which can be extracted from this task by subtracting performance on “recent positive” trials from “non-recent positive” trials. RT analyses were performed on trials with correct responses. Trials with RT over 3500 (0 instances) ms or under 100 ms (1 instance) were excluded as outliers (Greenberg et al. 2013).

### MRI acquisition and processing

Participants’ brains were scanned via a Siemans 3T scanner using a 32-channel head-coil. T1 weighted images were acquired via standard magnetization prepared rapid gradient-echo (MPRAGE) sequence (256 × 256 × 176, 1 mm isotropic voxels; TR = 2530 ms, TE = 1.69 ms, TI = 1100 ms, flip angle = 7°). FreeSurfer image analysis suite version 5.3 was used for cortical reconstruction and volumetric segmentation. To extract reliable volume estimates, images from before and after the training programs were automatically processed with the longitudinal stream (Reuter et al. 2012). Specifically an unbiased within-subject template space and image (Reuter and Fischl 2011) is created using robust, inverse consistent registration (Reuter et al. 2010). Several processing steps, such as skull stripping, Talairach transforms, and atlas registration as well as spherical surface maps and parcellations were then initialized with common information from the within-subject template, significantly increasing reliability and statistical power (Reuter et al. 2012). No manual interventions were needed following a visual check of segmentation accuracy. Using FreeSurefer’s “asegstats2table” and “long_stats_slopes”, we extracted volumes and symmetrized percent change of bilateral hippocampi in mm^3^ pre and post training programs were extracted using FreeSurefer’s “asegstats2table” and “long_stats_slopes”. Symmetrized percent change refers to the rate of change with respect to the average volume between two time-points rather than the rate of change from the first to the second time point. It has been shown to be a more robust and sensitive measure of longitudinal processing than standard percent change and other measures of longitudinal structure change (Reuter et al. 2012).

### Procedure

Following a phone screen to determine eligibility, participants were invited to the Martinos Center for Biomedical Imaging at Massachusetts General Hospital. Participants provided written informed consent, completed the Recent Probes task as well as other measures outside the scope of this report, and underwent a 9 min structural MRI scan. Participants started their training program within 0–2 weeks following completion of baseline testing. Testing procedures were repeated 0–2 weeks following the completion of the training programs.

### Statistical analysis

To assess group differences in proactive interference, one way Analyses of Covariance (ANCOVA) were performed on post-program proactive interference error rates and RT, adjusting for baseline rates, with group as the independent variable. Additionally, independent *t*-tests were conducted on change in proactive interference error rates and RT (calculated by subtracting baseline from post-program rates) to further probe group differences across time.

To assess group differences in hippocampal volume, independent *t*-tests of hippocampal symmetrized percent change (see *MRI acquisition and processing)* were conducted.

Following the per-protocol analyses described above, intent-to-treat analyses were performed by applying multiple imputations (Rubin 1996). Missing values were replaced by simulated values formed over 50 imputations and then pooled to produce a single result.

## Results

### Proactive interference

**Fig. 3:**
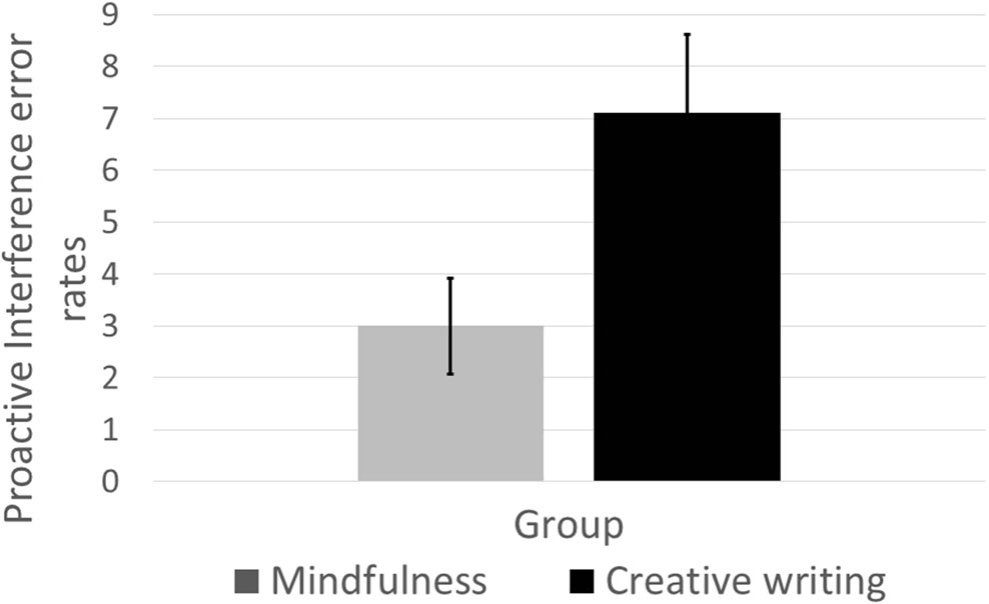
ANCOVA of post-program proactive interference error rates, adjusted for baseline error rates; *F*(1,51,) = 4.37, *p* = .04. Error bars represent standard errors

A *t*-test comparing baseline group performance from all participants with baseline data revealed that the mindfulness group exhibited significantly higher interference error rates compared to the control group (*t*(75) = 2.15, p = .034). No baseline differences were found in proactive interference RT (*t*(75) = 0.60, *p* = .55; see Table 2). To examine the effect of the programs on error rates while taking baseline performance into account, an ANCOVA with group as the independent variable was conducted on post-program proactive interference error rates, adjusting for baseline rates. A custom model including group, baseline error rates, and their interaction was examined to assess ANCOVA homogeneity assumptions, verifying that the homogeneity of regression slopes was met, as indicated by the non-significant interaction of group X baseline proactive interference error rates (*F*(1,50) = 0.66, *p* = .419). The homogeneity of variances assumptions was met as well, as indicated by non-significant of Levene’s test (*F*(1,52) = 0.44, *p* = .51). A full factorial model was then run. A significant effect was found for group, with the mindfulness group exhibiting a significantly lower proactive interference effect in error rates post-intervention compared to the active control group while controlling for baseline proactive interference error rates (*F*(1,51) = 4.37, *p* = .04, η_p_²=0.08; Fig. 3). To further assess whether groups differed across time, an independent *t*-test was conducted on the change in proactive interference error rates (see *Statistical W*). The test was found to be significant (*t*(52) = 3.29, *p* = .002) with the mindfulness group (*M* = .022, std. error = 0.012) showing greater improvement in proactive interference error rates compared to the active control group (*M*=-0.057, std. error = 0.0223). A similar *t*-test on all participants with available data regardless of adherence revealed a similar just-significant group difference (*t*(59) = 2.00, *p* = .05). Given that the data could be considered missing at random (see *Participants*), we then re-examined group differences in proactive interference using intent-to-treat analysis by applying multiple imputations (Rubin 1996). Missing values were replaced by simulated values formed over 50 imputations and then pooled to produce a single result. The *t*-test assessing group differences in change in proactive interference error rates using the pooled imputed values did not reach significance (*t*(445) = 1.38, *p* = .17).

**Table 2:** Main study variable comparison by group

	Baseline		Post-program		Longitudinal group difference
	Mindfulness	Control	Mindfulness	Control	
Proactive interference error rates	4.9% (1)	1.7% (0.8)	3% (1)	7.1% (1.7)	*F* = 4.37*, η_p_²=0.08
Proactive interference RT	121 (13)	109 (15)	119 (12)	112 (18)	*F* = 0.13
Left hippocampus volume (mm3)	4239.90 (70.24)	4188.55 (78.87)	4254.65 (77.56)	4040.35 (90.93)	*t* = 0.23
Right hippocampus volume (mm3)	4322.93 (63.59)	4311.06 (87.44)	4378.57 (72.35)	4130.00 (109.11)	*t* = 0.63

* p < .05. Baseline means are for all participants with available data. Standard Errors are shown in parentheses. Post scores for proactive interference are adjusted values following ANCOVA for participants completing the intervention. Longitudinal group differences are ANCOVA F values for proactive interference and t-values for group differences in symmetrized percent change for hippocampal volumes due its robustness for in longitudinal change

A similar ANCOVA as the one conducted on error rates was then conducted on post-program proactive interference RT, in which homogeneity assumptions were met (maximal *F* = 3.64, *p* = .06) The mindfulness group did not significantly differ from the active control group (*F*(1,51) = 0.13, *p* = .72; Table 2). Group differences in proactive interference RT remained non-significant when comparing groups’ change scores (*t*(52) = .97, *p* = .33), when including all participants with available data regardless of adherence (*t*(59) = .19, *p* = .85), and when comparing change scores with pooled imputed values in the intent-to-treat analysis described above (*t* = 0.10, *p* = .84). Neither group’s degree of home practice correlated with change in proactive interference measures (maximal *r*=-0.25, *p* = .12).

A secondary measure, though of lesser relevance to the current study can be extracted from the recent probes task - the degree to which old information can facilitate learning newer material (see *behavioral proactive interference task*). No differences were found between groups in ANCOVA run on facilitation in error rates or RT (maximal *F* = 0.63, *p* = .43).

### Hippocampal volume

For the subset of participants undergoing imaging (see *Participants*) there were no group differences in baseline hippocampal volume in either hemisphere, both when comparing participants’ original images (Table 2; maximal *t*(58) = 0.99, *p* = .33), and when comparing hippocampi volumes on the unbiased within-subject template space (Reuter and Fischl 2011; see MRI acquisition and processing section) for those in pre-post analysis (maximal *t*(44) = 1.84, *p* = .07). To test differences in hippocampal volume change following the programs, a *t*-test compared groups’ hippocampal symmetrized percent change (see *MRI acquisition and processing* for description) for each laterality. The mindfulness group (*M* = 0.27, *SD* = 2.57 for right hippocampus; *M* = 0.21, *SD* = 2.66 for left) did not differ from the active control group (*M*=-0.20, *SD* = 2.06 for right hippocampus; *M* = 0.02, *SD* = 2.35 for left) in symmetrized percent change for either laterality (maximal *t*(44) = 0.63, *p* = .53). Group differences remained non-significant when including all participants regardless of adherence (maximal *t*(49) = 0.68, *p* = .50), and following analysis of the pooled imputed values in intent-to-treat analysis (maximal *t* = .64, *p* = .52). The correlation between hippocampal symmetrized percent change and home practice time did not reach significance (maximal *r* = 0.50, *p* = .07).

### Relationship between improvement in proactive interference and hippocampal volume

A stepwise regression tested the relationship between improvements in proactive interference error rates in the mindfulness group and increases in hippocampal volume. Change in proactive interference error rates (calculated by subtracting post-program proactive interference error rates from those at baseline level) was the dependent variable, with symmetrized percent change in the left and right hippocampi as the predicting factors. A significant regression model was found with the left hippocampus as a predictor (*F*(1,28) = 6.36, *p* = .018), indicating that volume increase in the left hippocampus was significantly associated with improvements in proactive interference error rates (B = 0.012, Std Error = 0.005, β = 0.43). The multiple correlation coefficient was 0.43, indicating that overall volume increases in the left hippocampus explained 18.5% of the variance in proactive interference error rates improvement (*R*^2^ = 0.185). This correlation between improvements in proactive interference error rates and left hippocampal volume remained significant when including the imputed values in the intent-to-treat analysis (*F*(1,2528) = 141.21, *p* < .001; B = 0.008, Std Error = 0.001, β = 0.23).

A similar regression analysis conducted on the active control group alone did not yield a significant model (*F*(1,12) = 0.33, *p* = .57) and remained non-significant when including the imputed values in the intent-to-treat analysis (*F*(1,1367) = 1.20, *p* = .27). When adding group as an additional factor to the model, symmetrized percent change in the left hippocampus did not reach significance as a predictor (B = 0.007, Std. Error = 0.005, β = 0.22, *p* = .119), indicating that although the association between the change in hippocampus and proactive interference improvement was significant only for the mindfulness group, this association did not differ significantly between groups.

## Discussion

In the current study individuals undergoing mindfulness training exhibited significantly lower proactive interference error rates than the active control group, and these improvements were significantly associated with volume increases in the left hippocampus. These findings support the conceptualization of mindfulness as promoting attention to experience of the present moment while minimizing interference from past events.

Previous research has associated mindfulness training with functional and structural hippocampal changes (Gotink et al. 2016; Hölzel et al. 2011; Pickut et al. 2013; Wells et al. 2013). The current study is the first to link hippocampal change following mindfulness training to improvements memory performance. Furthermore, the association between improvements in proactive interference error rates and increases in hippocampal volume increases complements previous findings that relate hippocampal activity to decreased proactive interference error rates (Öztekin et al. 2008). This finding additionally provides novel evidence that this relationship is not limited to transient hippocampal functional activation during a task, but extends to longitudinal increases in hippocampal structure.

The left lateralization of this effect corresponds with our group’s previous work (Hölzel et al. 2011), which demonstrated structural increases specifically in the left hippocampus following mindfulness training. This left lateralization additionally supports numerous previous studies that indicate left hemispheric dominance during proactive interference (Badre and Wagner 2007; Jonides and Nee 2006; Nee et al. 2007; Öztekin et al. 2008).

No group differences were found in hippocampal volume following training. It is possible that this null effect is related to the relatively short duration of the training program. Previous mindfulness training studies examining subsequent hippocampal changes have used 8-week programs (Hölzel et al. 2011; Pickut et al. 2013; Wells et al. 2013), whereas the current study used a 4-week program. Additionally, home practice in the current study was limited to a maximum of 30 min 5 days a week in order to enable plausible future implementations of such training in the workplace, rather than the typically prescribed ~ 40 min 7 days per week for mindfulness based programs (Kabat-Zinn et al. 1985; Segal et al. 2012). It is possible that the structural hippocampal changes require a longer “dose” of practice.

Many populations that exhibit smaller hippocampal volumes also exhibit working memory related deficiencies, including healthy older adults (Bigler et al. 1997; Driscoll et al. 2003; Salthouse 1990, 1994), individuals who experienced childhood maltreatment or trauma (Majer et al. 2010; Stein et al. 1997), individuals suffering from depression (Drevets et al. 2008; Gotlib and Joormann 2010; Snyder 2012; Videbech and Ravnkilde 2004), and those with PTSD (Kitayama et al. 2005; Vasterling et al. 2002). Currently, mindfulness is being used primarily to treat distress and mood related symptoms. In light of the current results, it is possible that mindfulness may be particularly beneficial in the treatment of these conditions (see Kimbrough et al. 2010; Segal et al. 2012; Young and Baime 2010) in part due to the reduced memory interference and its association with increases in hippocampal volume.

Group differences in proactive interference were evident when applying per protocol analysis and did not reach significance when applying intent to treat analysis which replaces missing values with simulated values. However, the current study is mechanistic in nature. The National Institutes of Health propose a distinction between mechanistic studies and clinical efficacy trials (Lauer 2017) in terms of appropriate analysis plans. In light of this distinction we suggest that a better understanding of how the intervention works can be achieved by focusing on the participants who underwent the intervention.

Proactive interference is a central determinant of working memory capacity and functioning (Jonides and Nee 2006; Keppel and Underwood 1962). Working memory mediates higher order cognitive functions such as reasoning, learning, problem solving, language comprehension, and focusing attention on task-relevant information (Cowan 1995; Engle et al. 1999; Fry and Hale 1996; Jaeggi et al. 2008; Zanto and Gazzaley 2009). It is possible that the observed reduction in proactive interference in working memory following mindfulness training may in part underlie improvements in other reported cognitive improvements following mindfulness training which rely on working memory integrity, such as improvements in GRE scores (Mrazek et al. 2013), improved problem solving (Greenberg et al. 2012), increased sustained attention (Zeidan et al. 2010), and other cognitive capabilities (see Chiesa et al. 2011 for a review). Future studies may directly examine such mediation effects and, to maximize statistical power, recruit larger sample sizes and use longer programs than the present study.

## Conclusion

This study provides the first evidence that mindfulness training can reduce proactive interference in working memory – an ability essential for reasoning, learning, and problem solving – and that these improvements are associated with increases in the left hippocampal volume.
