# Correction to: Reduced interference in working memory following mindfulness training is associated with increases in hippocampal volume

**DOI:** 10.1007/s11682-018-9890-4

**Published:** 2018-05-15

**Authors:** Jonathan Greenberg, Victoria L. Romero, Seth Elkin-Frankston, Matthew A. Bezdek, Eric H. Schumacher, Sara W. Lazar

**Affiliations:** 1 Department of Psychiatry, Massachusetts General Hospital, Boston, MA USA; 2 Harvard Medical School, Boston, MA USA; 3 Charles River Analytics, Cambridge, MA USA; 4 School of Psychology, Georgia Institute of Technology, Atlanta, GA USA

**Correction to: Brain Imaging and Behavior**

10.1007/s11682-018-9858-4

The article Reduced interference in working memory following mindfulness training is associated with increases in hippocampal volume, written by Jonathan Greenberg, Victoria L. Romero, Seth Elkin-Frankston, Matthew A. Bezdek, Eric H. Schumacher, and Sara W. Lazar, was originally published electronically on the publisher’s internet portal (currently SpringerLink) on March 17, 2018 without open access.

With the author(s)’ decision to opt for Open Choice the copyright of the article changed on May 2018 to © The Author(s) 2018 and the article is forthwith distributed under the terms of the Creative Commons Attribution 4.0 International License (http://creativecommons.org/licenses/by/4.0/), which permits use, duplication, adaptation, distribution and reproduction in any medium or format, as long as you give appropriate credit to the original author(s) and the source, provide a link to the Creative Commons license and indicate if changes were made.

The original article has been corrected.

